# Ultrasound guided quadratus lumborum block versus interlaminar epidural block for analgesia in pediatric abdominal surgery: a randomized controlled trial

**DOI:** 10.1186/s12871-024-02548-z

**Published:** 2024-05-21

**Authors:** Mohamed Wageh, Mohamed Ahmed Sultan, Hazem El Sayed Moawad, Ehab Mohamed Mokbel, Mahmoud Mohammed Alseoudy

**Affiliations:** 1 Department of Anesthesia and Surgical Intensive care, Faculty of Medicine, Mansoura University, Mansoura, Egypt; 2 Abdelsalam Aref, St. Mansoura city, El- Dakahliya Governorate, Mansoura, Egypt

**Keywords:** Pediatric, Analgesia, Epidural block, Quadratus lumborum block, Abdominal surgery

## Abstract

**Background:**

Although the efficacy and safety of epidural block (EB) are fairly high, complications such as inadvertent dural puncture may limit its use. Ultrasound-guided quadratus lumborum block (QLB) is a relatively new regional technique that provides perioperative somatic and visceral analgesia for pediatric patients. This trial compared the quality of pain relief in pediatric patients undergoing abdominal surgery who received either QLB or EB.

**Methods:**

Patients were randomly allocated into two equal groups: Group E(*n* = 29): received EB; Group QL(*n* = 29): received QLB. Both groups were injected with 0.25% bupivacaine (0.5 ml/kg). Assessment of total analgesia consumption was the primary outcome measure, whereas the secondary outcome measures were assessment of postoperative analgesic effect by Children’s Hospital of Eastern Ontario Pain Scale (CHEOPS) and time of first analgesic request.

**Results:**

Our study showed that the mean total fentanyl consumption was comparable between both groups(38.67 ± 5.02 and 36.47 ± 5.13 µg in the E and QL groups, respectively, *P* = 0.246). Only five patients did not require rescue analgesia (3 in the E group,2 in the QL group, *P* = 0.378). The mean duration of analgesia showed no significant difference between the two groups (9.9 ± 1.58 and 11.02 ± 1.74 h in the E and QL groups, respectively, *P* = 0.212). Evaluation of CHEOPS score values immediately in PACU and for the initial 24 h following operation showed no significant difference between the two study groups(*P* > 0.05).

**Conclusion:**

QLB can achieve analgesic effects comparable to those of EB as a crucial part of multimodal analgesia in children undergoing abdominal surgeries.

**Clinical trial registration number:**

PACTR202203906027106.

## Introduction

A major contributor to the pain experienced by a patient after abdominal surgery is the incision made in the abdominal wall [1]. Almost 80% of patients undergoing surgery experience postoperative pain, and 80% of them reported moderate-to-severe pain intensity [2]. 

Failure to control postoperative pain causes significant clinical effects on the pediatric patient’s daily activities [3]. Children who experience significant postoperative pain experience slower recovery and increased postoperative morbidity, including poor oral intake, sleep disturbances, and behavioral changes [4]. This has led to regional analgesic approaches gaining tremendous popularity as crucial elements of postoperative analgesia regimens [5]. Evidence suggests that pain control achieved by regional blocks is at least comparable and, in many cases, superior to the intravenous techniques [6]. There is also greater hemodynamic stability, improved gastrointestinal function, less nausea and vomiting, and a reduced neurohumeral stress response [7]. Epidural block (EB) is a well-established and commonly performed neuraxial technique for providing intraoperative and postoperative analgesia to pediatric patients scheduled for lower abdominoperineal surgical interventions [8, 9]. Although the efficacy and safety of EB are fairly high [10], associated complications such as inadvertent dural puncture, unwarranted motor blockade of the lower limbs, and disturbance of bladder function might limit its use [11]. Ultrasound guided quadratus lumborum block (QLB) is a relatively new local anesthetic technique that provides perioperative somatic, perhaps even visceral, analgesia for patients of all ages, including pediatric patients, undergoing abdominal or hip surgery. The assumption is that a local anesthetic injected adjacently into the quadratus lumborum muscle will spread in a medial and cranial direction under the crura and arcuate ligaments of the diaphragm, and then into the thoracic paravertebral space (PVS) [12, 13]. In the current study, we hypothesized that QL block would be comparable to EB as an effective analgesia alternative with fewer side effects.

### Patients &methods

This prospective, randomized, double-blinded comparative study was conducted at Mansoura University Children’s Hospital (MUCH) between 2020 and 2022 after the approval of the Institutional Review Board (IRB) (IRB code number MS.20.06.1159). The study was registered on the Pan African Clinical Trials Registry (PACTR) (ID: PACTR202203906027106; date: 23/03/2022). Informed written consent was obtained from all patients’ parents. This trial was performed according to the ethical principles of the Declaration of Helsinki (2013) and was conduced in harmony with good clinical practice. Patients of both sexes aged between 2 and 7 years with American Society of Anesthesiologists (ASA) physical status I or II scheduled for abdominal surgery were included in the study. Patients with preexisting hepatic diseases, coagulation disorders, infection at the site of needle insertion, known allergies to bupivacaine, or parents’ refusal of consent were excluded from the study. All patients were subjected to preoperative assessment, and CHEOPS (14) was explained to all patients’ parents on the previous day of operation to cooperate with assessors to evaluate the postoperative pain score. CHEOPS includes six categories of pain behavior: cry, facial, verbal, torso, touch, and legs. Each is scored separately (ranging from 0 to 2 or 1–3) and calculated for a pain score ranging from 4 to 13, with a minimum score of 4 points (no pain) and the maximum being 13 points (most awful pain). After application of standard anesthesia monitoring (electrocardiogram, non-invasive blood pressure, oxygen saturation), general anesthesia was induced with propofol (2 mg/kg), fentanyl (1 µg/kg) and atracurium (0.5 mg/kg for intubation and 0.1 mg/kg for maintenance as needed). An endotracheal tube was used to secure the airway. Anesthesia was maintained using 2% sevoflurane in a 50% air-oxygen mixture. The children were randomly allocated to either the epidural group (group E ) or the quadratus lumborum group (group QL). Group E (*N* = 29 patients) received 0.5 ml/kg of 0.25% bupivacaine for epidural block. Group QL: (*N* = 29 patients) received 0.5 ml/kg of 0.25% bupivacaine for quadratus lumborum block. Randomization concealment was performed using opaque sealed envelopes. The patient identifiers were attached to the opened envelopes and secured by a dedicated person who was independent of the randomization proceedings. This was a double-blinded study in which both anesthetist responsible for data collection and responsible patients’ parents included in the study were blinded to group allocation. All patients were positioned in the lateral position with the hips fully flexed, sterilized, and covered with sterile sheets. Aseptic precautions were taken by wearing sterile gowns and gloves. A utrasound machine (VINNO Technology Co., Ltd., Suzhou, China) with a high-frequency (6.5–18 MHz) linear transducer (X6–16 L) was used. A uniform dressing for all patients after injection, regardless of the group, was used to maintain the blindness of the type of intervention.

### Ultrasound guided epidural block technique

**Fig. 1:**
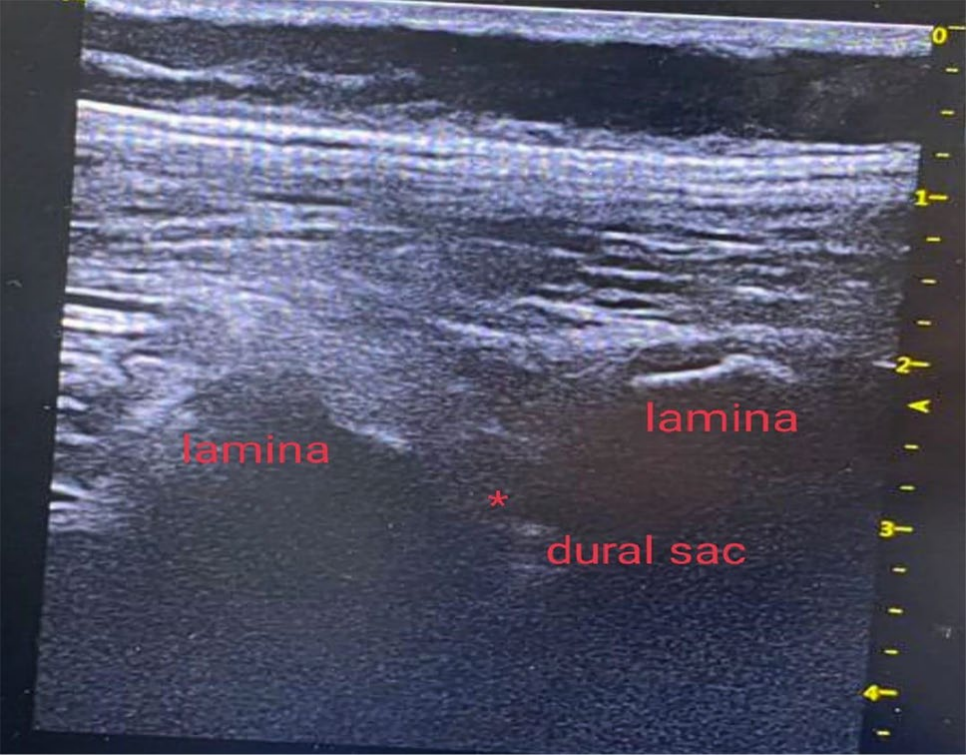
Ultrasound guided epidural block. _*_: site of injection

A high-frequency probe was used to scan from the sacral to the thoracic level and to confirm the target level. After selecting a puncture site (T12-L1), the distance from the skin to the ligamentum flavum was measured in the paramedian view to serve as a reference during needle insertion. Following sterile preparation, the assistant positioned the US probe superior to the puncture site in the paramedian plane to visualize the hyperechoic dura mater and ligamentum flavum, enabling free use of both hands by the operator. The needle was angulated in a cephalad direction to locate the needle tip within the epidural space (Fig. 1). When the needle tip was observed to pass through the ligamentum flavum and enter the epidural space on US images, its position was confirmed by the air-loss of resistance (LOR) technique. After the LOR test, when a small volume of saline or local anesthetic was injected, ventral displacement of the dura mater and widening of the epidural space could be observed on US images. We then injected 0.5 ml/kg bupivacaine 0.25%.

### Ultrasound guided quadratus lumborum block technique

**Fig. 2:**
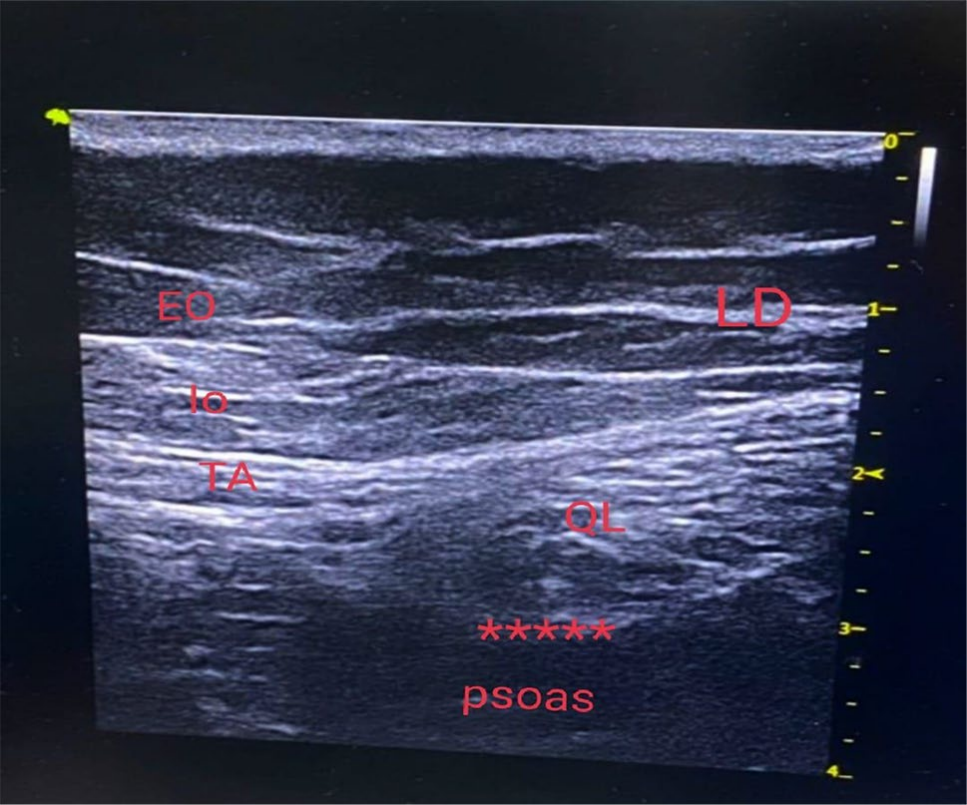
Ultrasound guided quadratus lumborum block. _*****_: site of injection. QL: quadratus lumborum; EO: externak oblique; IO: internal oblique; TA: transversus abdominis; LD: latismus dorsi

The probe was placed in the mid-axillary line cranially to the iliac crest to identify the three muscles of the anterior abdominal wall (transversus abdominis, internal oblique, and external oblique), then scanned dorsally, keeping the transverse orientation, until it was observed that the transversus abdominis muscle became aponeurotic. This aponeurosis was followed until the QL muscle was clearly visualized with its attachment to the lateral edge of the transverse process of L4 and psoas muscle. The needle was inserted in-plane from anterior to posterior, and its tip was advanced toward the anterior border of the QL muscle. Between the QL and psoas muscles, a 1 ml test dose of saline was injected to confirm the correct needle-tip position, and then this was followed by an injection of 0.5 ml/kg of 0.25% bupivacaine (Fig. 2). Bilateral injection was performed for midline incisions and unilateral injection for paramedian incisions. At the end of surgery, inhalational anesthesia was discontinued, and neuromuscular block was reversed with neostigmine (0.04 mg/kg) and atropine (0.02 mg/kg). Extubation was performed when patients had fulfilled the required criteria, and then patients were transferred to the post-anesthesia care unit (PACU). All patients received postoperative paracetamol intravenously (i.v.) as routine analgesia (10 mg/kg/8 h) considering the first dose at the end of surgery. Fentanyl i.v. (1 µg/kg/dose) was given as rescue analgesia for patients in the two study groups if the CHEOPS pain score ≥ 5. Assessment of the postoperative total fentanyl consumption was the primary outcome, while the secondary outcomes were the assessment of postoperative pain by the Children’s Hospital of Eastern Ontario Pain Scale (CHEOPS) 0 (immediate postoperative), 6, 12, 18, and 24 h post-operatively., the time of the first analgesic request, the occurrence of complications such as nausea, vomiting, and dural puncture, and the patient’s parents’ satisfaction regarding the analgesia.

### Sample size calculation and statistical analysis

Sample size was calculated using G*Power software version 3.1.9.7, using the results published by Ipek et al. (15) comparing the rescue analgesia requirement dosage in pediatric patients receiving either caudal epidural block or quadratus lumborum block for lower abdominal surgery under general anesthesia as the primary outcome. A sample size of 23 patients in each group was needed to achieve 90% power using a two-sided, two-sample equal-variance t-test with a significance level of (α 0.05). The total number was increased to 30 patients per group to compensate for possible dropouts. IBM’s SPSS statistics (Statistical Package for the Social Sciences) for Windows (version 22) were used for statistical analysis of the collected data. The Shapiro-Wilk test was used to check the normality of the data distribution. Normally distributed continuous variables were expressed as mean ± SD, while categorical variables and the non-normally distributed continuous ones were expressed as median and range, or number and percentage (as appropriate). The Student t test and Mann-Whitney were used for normally and non-normally distributed continuous data, respectively. The chi square test was used for categorical data using the crosstabs analysis. All tests were conducted with a 95% confidence interval. A P (probability) value < 0.05 was considered statistically significant.

## Results

**Fig. 3:**
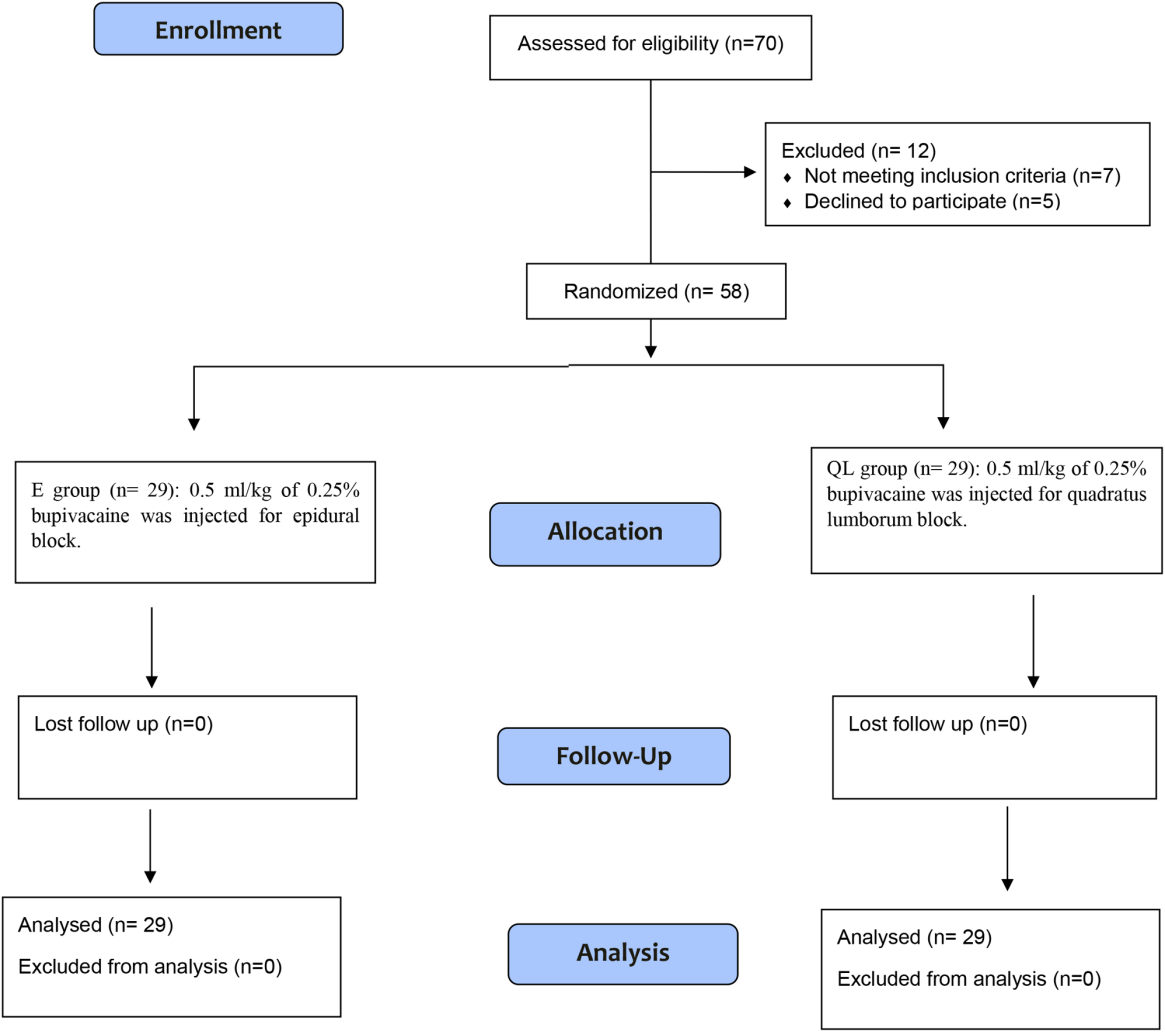
Consort flow diagram showing recruitment of patients in the study. E: epidural, QL: quadratus lumborum

**Fig. 4:**
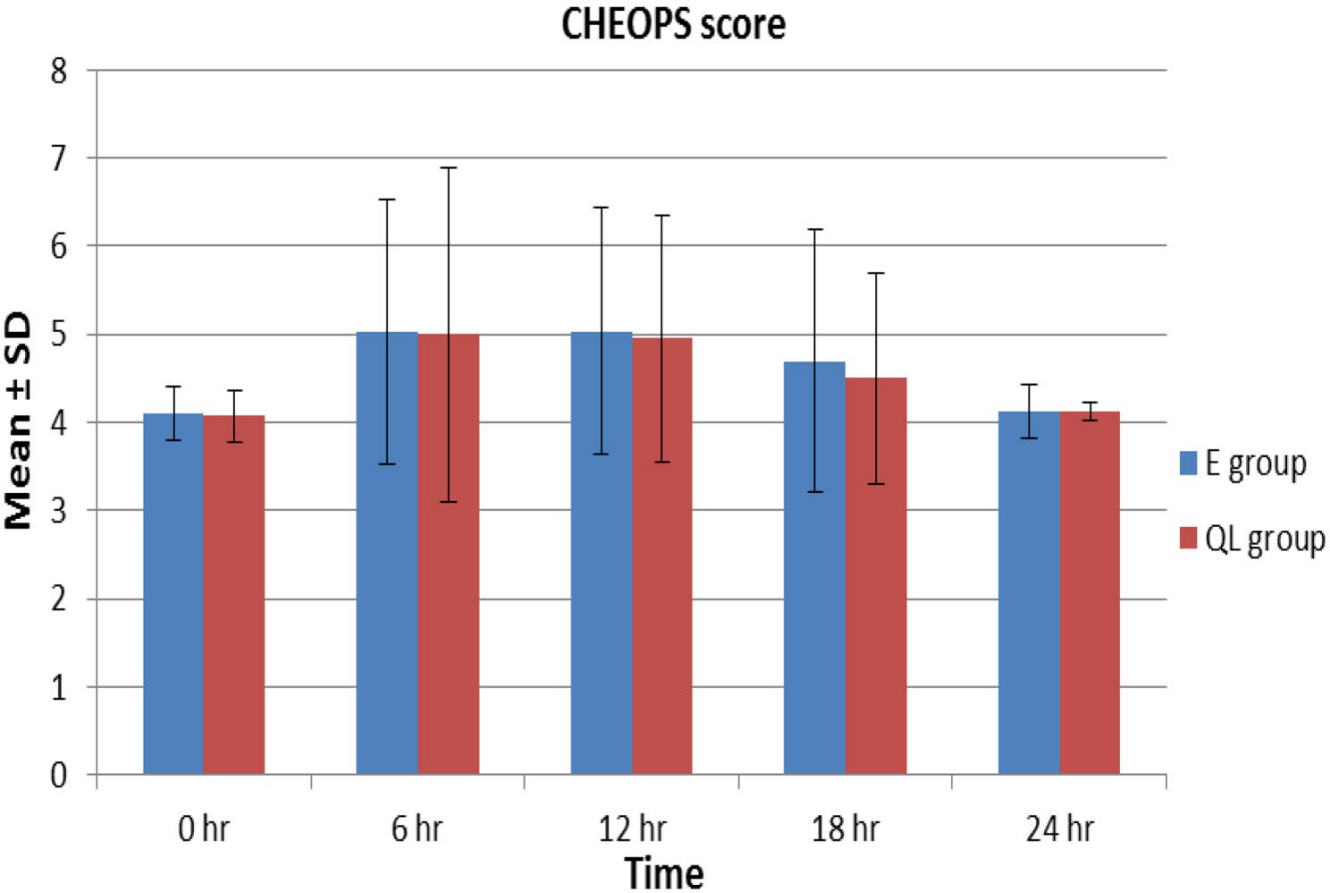
Follow up of CHEOPS score in the two study groups. Data are expressed as mean ± SD. *E: Epidural, QL: Quadratus lumborum. 0 h means immediate postoperative in PACU*

This randomized controlled, double-blind study assessed the eligibility of seventy patients. Twelve patients were excluded (7 did not fulfill criteria; 5 refused to participate). The remaining 58 patients were randomized into two groups (29 each), the E group and the QL group (Fig. 3). The patients were comparable in terms of their characteristics, type of surgery, duration of anesthesia, and duration of operation (Table 1). The assessed parameters for postoperative analgesia (Table 2) were comparable in both groups. The duration of analgesia showed no significant difference between the two groups (9.9 ± 1.58 and 11.02 ± 1.74 h in the E and QL groups, respectively, *P* = 0.212). Likewise, the total fentanyl consumption was comparable between both groups (38.67 ± 5.02 and 36.47 ± 5.13 µg in the E and QL groups, respectively, *P* = 0.246). Only five patients did not require rescue analgesia (3 in the E group, 2 in the QL group, *P* = 0.378). Evaluation of CHEOPS score values immediately in PACU and for the initial 24 h following operation showed no statistically significant difference between the two study groups (*P* > 0.05) (Fig. 4). Generally, parents` satisfaction showed no statistically significant difference on statistical analysis (*P* = 0.086). However, 82.6% of QL group patients were satisfied compared to only 72.4% of cases in the E group (Table 3). There was no recorded intraoperative bradycardia or hypotension in either group. With regard to postoperative complications, vomiting was reported by 6.9% and 10.3% in the E and QL groups, respectively. Dural puncture was encountered in only 6.9% of patients in the E group (6.9%). No significant difference was noted between the groups regarding the incidence of complications (*P* = 0.146). (Table 3)

**Table 1:** Characteristics of patients and surgery for both groups

Variable	E group (*n* = 29)	QL group (*n* = 29)	*P*-value
Age (years)	4.7 ± 1.3	4.7 ± 1.2	0.862
**Sex**			
Male	12 (41.4%)	14 (48.3%)	0.354
Female	17 (58.6%)	15 (51.7%)	
Weight (Kg)	15.5 ± 2.9	15.4 ± 2.6	0.464
**ASA score**			
I	27 (93.1%)	29 (100%)	0.642
II	2 (6.9%)	0 (0%)	
**Type of surgery**			
Appendectomy	11 (37.9%)	12 (41.4%)	0.892
Nephrectomy	3 (10.3%)	3 (10.3%)	
Excision of abdominal mass	8 (27.6%)	7 (24.1%)	
Closure colostomy	2 (6.9%)	3 (10.3%)	
Incisional hernia repair	2 (6.9%)	2 (6.9%)	
Splenectomy	3 (10.3%)	2 (6.9%)	
Duration of operation (min)	64.26 ± 4.11	67.08 ± 5.15	0.514
Duration of anesthesia (min)	77.6 ± 5.8	77.4 ± 3.8	0.486

Data are expressed as mean ± SD and number(%)

E: Epidural, QL: Quadratus lumborum

**Table 2:** Assessed parameters for post-operative analgesia in the two study groups

Variable	E group (*n* = 29)	QL group (*n* = 29)	*P*-value
Duration of analgesia (hours)	9.9 ± 1.58	11.02 ± 1.74	0.212
Total 24 h postoperative dose of fentanyl (µg)	38.67 ± 5.02	36.47 ± 5.13	0.246
Number of patients without need of rescue analgesia	3 (10.3%)	2 (6.9%)	0.378

Data expressed as Number(%)

*: significant (*p* < 0.05).E: Epidural, QL: Quadratus lumborum

**Table 3:** Satisfaction of the cases` parents and recorded complications within the study groups

Variable	E group (*n* = 29)	QL group (*n* = 29)	*P*-value
**Satisfaction**			
Satisfied	21 (72.4%)	24 (82.6%)	**0.086**
Not satisfied	8 (27.6%)	5 (17.4%)	
**Complications**			
Vomiting	2 (6.9%)	3 (10.3%)	**0.146**
Dural puncture	2 (6.9%)	0 (0%)	

Data are expressed as Number (%). E: Epidural, QL: Quadratus lumborum

## Discussion

To the best of our knowledge, this is the first randomized, double-blind, controlled study comparing the analgesic effects of interlaminar epidural block and quadratus lumborum block in children undergoing abdominal surgery. Our study demonstrated that quadratus lumborum block can provide postoperative analgesia in children undergoing major abdominal surgery with a quality comparable to epidural block because we found no statistically significant difference between both blocks regarding time of rescue analgesia, total postoperative fentanyl consumption, number of patients with no need for rescue analgesia and post-operative CHEOPS values. An abdominal truncal block known as the QL block has emerged, and is applied for somatic analgesia in both the lower and upper abdomen. With the QL block, it is possible for the local anesthetic to spread from the posterior aspect of the quadratus muscles to the thoracolumbar fascia’s medial layer, situated closer to the thoracic paravertebral space [14–17]. Ultrasound-guided QL block was first described by Rafael Blanco [18], who clarified that QL block differs from the known TAP block (Transversus Abdominis Plane block) as the latter is superficial to the transversus abdominis muscle and its aponeurosis, while the QLB is actually deep to the transversus abdominis aponeurosis [19]. In the QLB, LA spreads from its lumbar deposition cranially into TPVS; this could explain why QLB seems to be able to alleviate both somatic and visceral pain [20, 21] and why QLB could provide analgesia after abdominal surgeries [12, 22]. Blanco and colleagues showed that the QL block was superior to the TAP block concerning the duration of analgesia and opioid consumption. TAP block can cover dermatomes from T10 to T12 whereas the QL block can affect dermatomes from T7 to T12. This extensive spread with QL block could produce analgesia for somatic and visceral pain [23]. Our study results detected a comparable analgesic effect of QL block and epidural block, which was supported by Ipek et al., who included 94 children randomly divided into three equal groups to perform TAP, QL, or caudal epidural block using 0.25% of bupivacaine solution (0.5 ml/kg). The authors showed that the first analgesic request time, number of patients who required analgesia in the first 24 h postoperatively, and fentanyl requirements did not differ among the three groups, concluding that ultrasound-guided QL block could be considered a good option for perioperative analgesia in pediatric patients undergoing lower abdominal surgery [15]. Our results were in accordance with those of Aksu C and Gürkan Y, who initiated ultrasound-guided QL block to provide postoperative analgesia for ambulatory inguinal hernia repair surgeries in pediatric anesthesia practice. They presented results from their first 10 patients. The patients were observed to be relaxed and calm in the postoperative care unit. No patients required additional analgesics. The patients were discharged from the hospital at the postoperative fourth and fifth hours. They encouraged controlled studies involving sufficient patients to detect the distribution of local anesthetics and the field of coverage [24]. Similar to our results, Sabra and Abotaleb showed that there was no statistically significant difference in the CHEPOS score or complications (hemodynamic instability, injury to underlying structures, hematoma formation, infection, and postoperative nausea and vomiting) between pediatric patients who received either QL block or caudal epidural block during unilateral lower abdominal surgery [25]. However, the authors showed higher patient parents’ satisfaction (*P* < 0.001) in the QL group than in the caudal epidural and control groups. Sato included 44 pediatric patients aged between 1 and 17 years undergoing bilateral ureteral re-implantation surgery via a low transverse incision, randomized into quadratus lumborum block and caudal block groups. He found that fentanyl requirements for postoperative rescue analgesia during the first 24 h were significantly lower in the QL block group than in the caudal block group ((*p* = 0.016), with comparable CHEOPS values, and no postoperative complication (hypotension, arrhythmia, or bradycardia) was observed in any of the patients [26]. He performed quadratus lumborum block type 2 (QLB-2) where the local anesthetic was injected between the posterior border of the quadratus lumborum muscle and thoracolumbar fascia, separating it from the latissimus dorsi and paraspinal muscles. In our study, we performed quadratus lumborum block type 3 (QLB-3) where the local anesthestic was injected between the anterior surface of the QLM and psoas major muscles. A recent study conducted by Yetik et al. to compare the postoperative analgesic effects of ultrasound-guided QLB-2 and QLB-3 after cesarean section under general anesthesia demonstrated that pain scores and postoperative tramadol consumption were statistically lower in the QLB-3 group than in QLB-2 [27]. In a recent meta-analysis conducted by Zhao et al. to evaluate the postoperative analgesic effect of QL block in pediatric patients undergoing lower abdominal surgeries, their results showed that the rate of postoperative rescue analgesia was significantly lower in the QL group than in other analgesic groups (caudal, TAP, and ESPB). QL block might also reduce pain scores after surgery without increasing adverse events compared with other analgesic groups [28]. In the current study, the frequency of vomiting was 6.9% and 10.3% in the epidural and QL groups, respectively. No statistically significant difference was noted between the two groups. The low incidence of emesis in the two groups may be due to the use of propofol as an intraoperative anesthetic. Dural puncture occurred in two patients from the 29 patients included, whereas in the study of Tanya Mital et al. [29], it occurred in none of the patients in the ultrasound-guided group from 23 patients and four patients in the landmark-guided group from 22 patients. This result reflects an increased margin of safety for pediatric epidurals with the use of ultrasound.

Although we performed regional blocks in different types of abdominal surgeries with potential variability in surgical aggressiveness, which may be a limitation of our study, it may have the advantage of adequate randomization and generalizability of applicable results.

This study had some limitations. First, a small sample size may underpower the study to detect differences in complications. Second, we did not measure the effectiveness of the two interventions in relation to placebo or sham control group. Third, we did assess the sensory dermatome block level because the techniques were performed after the induction of general anesthesia. Future studies may be conducted to assess the efficacy of QLB using different local anesthetics and adjuvants such as magnesium sulfate and dexmedetomidine, assess the feasibility of QLB for providing complete surgical anesthesia for abdominal surgeries in children, and evaluate the efficacy of using continuous analgesia by catheter insertion for EB and QLB not only single shots.

In conclusion, QLB can achieve analgesic effects comparable to those of EB as a crucial part of multimodal analgesia in children undergoing abdominal surgeries.

## Data Availability

The datasets used and analyzed during the current study are available from the corresponding author on reasonable request. Mahmoud Mohammed Alseoudy; e-mail drs3ody.mansora@mans.edu.eg

## Abbreviations

QLB: quadratus lumborum block
EB: epidural block
CHEOPS: Children’s Hospital of Eastern Ontario Pain Scale (CHEOPS)
SPSS: Statistical Package for the Social Sciences
PVS: paravertebral space
LOR: loss of resistance
